# COVID-19 mortality in the UK Biobank cohort: revisiting and evaluating risk factors

**DOI:** 10.1007/s10654-021-00722-y

**Published:** 2021-02-15

**Authors:** Joshua Elliott, Barbara Bodinier, Matthew Whitaker, Cyrille Delpierre, Roel Vermeulen, Ioanna Tzoulaki, Paul Elliott, Marc Chadeau-Hyam

**Affiliations:** 1 Department of Epidemiology and Biostatistics, School of Public Health, Imperial College London, St Mary’s Hospital, Norfolk Place, London, W21PG UK; 2 MRC Centre for Environment and Health, Imperial College, London, UK; 3 Royal Surrey County Hospital, Guildford, Surrey, GU2 7XX UK; 4 UMR LEASP, Université de Toulouse III, UPS, Inserm, Toulouse, France; 5 Department of Hygiene and Epidemiology, University of Ioannina Medical School, Ioannina, Greece; 6 Institute for Risk Assessment Sciences (IRAS), Utrecht University, Utrecht, The Netherlands

**Keywords:** COVID-19 mortality, SARS-CoV-2, Prospective cohort, UK biobank, Risk factor

## Abstract

Most studies of severe/fatal COVID-19 risk have used routine/hospitalisation data without detailed pre-morbid characterisation. Using the community-based UK Biobank cohort, we investigate risk factors for COVID-19 mortality in comparison with non-COVID-19 mortality. We investigated demographic, social (education, income, housing, employment), lifestyle (smoking, drinking, body mass index), biological (lipids, cystatin C, vitamin D), medical (comorbidities, medications) and environmental (air pollution) data from UK Biobank (N = 473,550) in relation to 459 COVID-19 and 2626 non-COVID-19 deaths to 21 September 2020. We used univariate, multivariable and penalised regression models. Age (OR = 2.76 [2.18–3.49] per S.D. [8.1 years], *p* = 2.6 × 10^–17^), male sex (OR = 1.47 [1.26–1.73], *p* = 1.3 × 10^–6^) and Black versus White ethnicity (OR = 1.21 [1.12–1.29], *p* = 3.0 × 10^–7^) were independently associated with and jointly explanatory of (area under receiver operating characteristic curve, AUC = 0.79) increased risk of COVID-19 mortality. In multivariable regression, alongside demographic covariates, being a healthcare worker, current smoker, having cardiovascular disease, hypertension, diabetes, autoimmune disease, and oral steroid use at enrolment were independently associated with COVID-19 mortality. Penalised regression models selected income, cardiovascular disease, hypertension, diabetes, cystatin C, and oral steroid use as jointly contributing to COVID-19 mortality risk; Black ethnicity, hypertension and oral steroid use contributed to COVID-19 but not non-COVID-19 mortality. Age, male sex and Black ethnicity, as well as comorbidities and oral steroid use at enrolment were associated with increased risk of COVID-19 death. Our results suggest that previously reported associations of COVID-19 mortality with body mass index, low vitamin D, air pollutants, renin–angiotensin–aldosterone system inhibitors may be explained by the aforementioned factors.

**Supplementary Information:**

The online version contains supplementary material available at 10.1007/s10654-021-00722-y.

## Introduction

Coronavirus disease 2019 (COVID-19) was first documented in the UK at the end of January 2020, with possible community transmission likely to have started earlier [1]. On 11 March 2020, the World Health Organization classified COVID-19 as a global pandemic [2]. As of 18 December 2020, more than 1,600,000 deaths globally had been attributed to COVID-19 [3], with over 60,000 deaths in the UK [4].

There is accumulating evidence that older age, male sex and non-White ethnicity are key risk factors for severe or fatal COVID-19 [5, 6]. Additionally, a range of comorbidities have been implicated in COVID-19 risk, including hypertension [7], cardiovascular disease [8], kidney disease [9] and diabetes [10–13]. There is also interest in the role of lifestyle and environmental factors such as obesity [14], smoking [15], vitamin D [16, 17] and air pollutants [18]. Some medications are also theorised to affect risk such as inhibitors of the renin–angiotensin–aldosterone system (RAAS), including angiotensin-converting-enzyme inhibitors (ACEi) or angiotensin II receptor blockers (ARB) [11–13, 19, 20], as well as long-term systemic steroid (glucocorticoid) use [21] and statin therapy [22–24].

Much of the research to date has relied on routine clinical data that are prone to a range of biases, in particular selection bias due to hospitalised cases being more severe and not representative of the disease burden in the community [25, 26]. Additionally, there are differences in study design and population characteristics that may have resulted in inconsistencies between studies [11, 27–30]. UK Biobank offers the benefit of detailed baseline participant characterisation and a community-based sample.

In the present work, we investigate risk factors for COVID-19 and non-COVID-19 death since January 2020 using the latest mortality data linked to UK Biobank (to 21 September 2020) and quantify their independent and joint contribution to COVID-19 mortality through sequential adjustment and variable selection approaches.

## Study and methods

### Study population

UK Biobank is a population-based cohort of 502,506 volunteers (5.5% response rate) [31] with current consent, aged 40 to 69 years at recruitment from 2006 to 2010. There were 28,956 deaths up to 31 January 2020—the date of the first recorded UK COVID-19 case—leaving N = 473,550 for the present study, among whom there have been 459 COVID-19 deaths and 2626 non-COVID-19 deaths as of 21 September 2020. These deaths were recorded through linkage to national death registries (NHS Digital, NHS Central Register, National Records of Scotland). The ICD-10 codes denoting COVID-19 death were U07.1 (N = 438, virus identified in laboratory testing) and U07.2 (N = 21, clinical or epidemiological diagnosis of COVID-19 where laboratory testing was inconclusive or not available). At enrolment, participants completed a touch screen questionnaire and provided a blood sample analysed for biochemical and haematological markers.

### Participant characteristics

We considered six categories of variables potentially associated with COVID-19 mortality: demographic, social, health risk, biological, medical, and environmental factors [32] (Supplementary Methods). Demographic variables were age, sex and ethnicity (White, Black, Other). Social variables were educational attainment, housing, average household income and occupation. Educational attainment was categorised as high (College or University degree), intermediate (A/AS levels, O levels/General Certificate of Secondary Education (GCSE), Certificate of Secondary Education (CSE), National Vocational Qualification (NVQ) or Higher National Diploma (HND), or equivalent, and other professional qualifications) and low (none of the above). Housing was characterised by (i) type of accommodation (house/bungalow or flat), (ii) whether the accommodation was rented, owned outright or owned with a mortgage, and (iii) number of individuals living in household. Average household income was categorised as: less than GBP 18,000; GBP 18,000–30,999; GBP 31,000–51,999; more than GBP 52,000. Occupation at recruitment was coded as employed healthcare workers, employed non-healthcare workers, unemployed and retired. We included five biochemical markers: lipids (total cholesterol [mmol/L], high density lipoprotein cholesterol [HDL, mmol/L], triglycerides [mmol/L]), vitamin D (nmol/L), and cystatin C (mg/L) as a marker of renal function [33]. Health risk factors were smoking and alcohol drinking status (current, former, never) and body mass index (BMI): < 25, 25–30, 30–40 and > 40 kg/m^2^. Medical factors included six comorbidities (cancer, cardiovascular disease, hypertension, diabetes, respiratory disease and autoimmune disease) based on self-reported information at enrolment and via linkage to Hospital Episode Statistics in England and the equivalent in Scotland and Wales. Additionally, baseline glycated haemoglobin level ≥ 48 mmol/mol was used to classify diabetes (Supplementary Table 1A). We also included use of ACEi, ARB, oral steroid or statin as reported at enrolment (see detailed codes in Supplementary Table 1B). Environmental exposures were modelled levels of nitrogen oxides (NO_x_) and particulate matter (PM10, PM2.5 and PM2.5 absorbance) at residential address in 2010 [34].

### Statistical analyses

We compared means, proportions and estimated odds ratios (ORs) from univariate logistic regression for each covariate in all (N = 459) participants who died from COVID-19 or other causes (N = 2626) versus those alive (N = 470,465) from 31 January to 21 September 2020. Continuous variables were standardised so that ORs were expressed on comparable scales per standard deviation increase (8.09 years for age, 1.14 mmol/L for cholesterol, 0.38 mmol/L for HDL cholesterol, 1.02 mmol/L for triglycerides, 21.07 mmol/L for vitamin D, 0.16 mg/L for cystatin C, 15.50 ug/m^3^ for NO_x_, 1.90 ug/m^3^ for PM_10_, 0.27 absorbance/m for PM_2.5_ absorbance, and 1.06 ug/m^3^ for PM_2.5_).

To estimate the mutually adjusted effect size estimates of the variables under investigation, we sequentially adjusted logistic models for time-resolved covariates. Specifically, our benchmark model was adjusted for age, sex and ethnicity. Our analyses were subsequently adjusted for (i) social factors; (ii) health risk factors; (iii) biological factors; (iv) medical variables (comorbidities and medications) and (v) environmental factors. As a complementary analysis accounting for correlation between covariates, we used logistic LASSO (penalised) regression. This approach aimed to identify a parsimonious set of variables jointly explaining risk of COVID-19 or non-COVID-19 death, as well as estimating their joint (and mutually-adjusted) effects [35]. These were calibrated using tenfold cross-validation minimising the binomial deviance. In order to assess if the set of selected variables might have been driven by outlying observations, we investigated the stability of the variable selection by fitting logistic LASSO models on (N = 1000) random 80% subsamples of the study population. Each subsample included the same proportion of COVID-19 and non-COVID-19 deaths representative of that observed in the full UK Biobank sample. We report selection proportion as a measure of relevance for each variable.

In order to quantify and compare the mortality-relevant information from different sets of predictors across models, we conducted a series of receiver operating characteristic (ROC) analyses. Over 1000 iterations, we used 80% subsamples as training sets and calculated the area under the ROC curve (AUC) in the remaining 20% test sets.

All analyses were performed in R, version 4.0.2.

## Results

### Descriptive statistics and univariate analyses

Between 31 January and 21 September 2020, a total of 3085 deaths were recorded in UK Biobank, of which 459 (14.9%) were coded as COVID-19 deaths. Descriptive statistics and results of univariate logistic models are given in Table 1, Supplementary Figure 1, and Supplementary Table 2. For the 459 COVID-19 deaths, mean age was 6.6 years greater than the remaining cohort; comparison of characteristics for deaths assigned to different COVID-19 ICD codes is given in Supplementary Table 3. Risk of COVID-19 death was higher in older individuals (OR = 3.0 [2.63–3.43] for an increase of 8.1 years, *p* = 7.24 × 10^–60^), men (OR = 2.15 [1.78–2.60], *p* = 3.3 × 10^–15^), participants of Black ethnicity (OR = 3.17 [2.08–4.82], *p* = 7.7 × 10^–8^) and those with comorbidities (OR ≥ 1.73, *p* ≤ 5.7 × 10^–7^). In addition, there was higher risk in participants of low and intermediate educational attainment, low earners, healthcare workers, unemployed and retired people, those renting, living in a flat and with lower mean number of people per household (OR ≥ 1.43, *p* ≤ 5.4 × 10^–3^). Risk of COVID-19 death was also higher among former and current smokers, former and never drinkers, overweight, obese and morbidly obese participants (OR ≥ 1.66, *p* ≤ 9.3 × 10^–5^) as recorded at enrolment. Risk was higher in those with higher levels of triglycerides and cystatin C (OR ≥ 1.16, *p* ≤ 2.7 × 10^–4^); lower cholesterol, HDL, and vitamin D (OR ≤ 0.87, *p* ≤ 9.3 × 10^–3^); in participants taking an ACEi, ARB, oral steroids, or a statin at enrolment (OR ≥ 2.41, *p* ≤ 1.51 × 10^–7^); and those exposed to higher levels of air pollution at residence (OR ≥ 1.14, *p* ≤ 4.8 × 10^–3^). These variables, except Black ethnicity, healthcare worker status, and higher levels of PM_2.5_ (absorbance) and PM_10_ were also associated with higher risk of non-COVID-19 mortality (Supplementary Figure 1, Supplementary Table 2). Comparison of results from univariate regression models for deaths assigned to different COVID-19 ICD codes is given in Supplementary Figure 2.

**Table 1:** Characteristics of the UK Biobank study population: participants who were, alive, dead from COVID-19 or dead from another cause than COVID-19 as of 21 September, 2020 in the full UK Biobank sample

	Alive (N = 470,465)		COVID-19 deaths (N = 459)		Non-COVID-19 deaths (N = 2626)	
	N	Mean (SD)/Proportion	N	Mean (SD)/Proportion	N	Mean (SD)/Proportion
**Demographics**						
Age (years)	470,465	67.86 (8.09)	459	74.50 (5.96)	2626	73.82 (6.26)
*Sex*						
Women	260,378	55.34%	168	36.60%	1158	44.10%
Men	210,087	44.66%	291	63.40%	1468	55.90%
*Ethnicity*						
White	442,001	94.46%	415	91.41%	2529	96.75%
Black	7734	1.65%	23	5.07%	27	1.03%
Other	18,175	3.88%	16	3.52%	58	2.22%
**Social**						
*Education*						
High	153,845	33.35%	87	19.91%	634	24.67%
Intermediate	231,765	50.24%	188	43.02%	1162	45.21%
Low	75,665	16.40%	162	37.07%	774	30.12%
*Type of accommodation*						
House	420,969	90.26%	369	82.92%	2246	86.89%
Flat	45,426	9.74%	76	17.08%	339	13.11%
*Own or rent accommodation*						
Own outright	239,996	52.24%	258	58.64%	1655	65.21%
Own with a mortgage	176,654	38.45%	91	20.68%	503	19.82%
Rent	42,734	9.30%	91	20.68%	380	14.97%
Number in household	466,469	2.46 (1.32)	443	2.06 (1.03)	2589	2.06 (1.16)
*Average household income (GBP)*						
Less than 18,000	86,640	21.69%	171	46.85%	839	39.33%
18,000 to 30,999	100,837	25.24%	93	25.48%	633	29.68%
31,000 to 51,999	106,021	26.54%	62	16.99%	378	17.72%
Greater than 52,000	106,008	26.53%	39	10.68%	283	13.27%
*Occupation*						
Unemployed	64,136	13.79%	78	17.18%	423	16.25%
Employed (Healthcare worker)	27,925	6.00%	20	4.41%	84	3.23%
Employed (Other)	236,502	50.85%	102	22.47%	697	26.78%
Retired	136,489	29.35%	254	55.95%	1399	53.75%
**Health risk factors**						
*Smoking status*						
Never	261,361	55.87%	171	37.75%	1111	42.68%
Former	159,706	34.14%	211	46.58%	1052	40.41%
Current	46,745	9.99%	71	15.67%	440	16.90%
*Alcohol drinker status*						
Never	20,762	4.43%	35	7.69%	168	6.42%
Former	15,938	3.40%	34	7.47%	138	5.28%
Current	432,254	92.17%	386	84.84%	2309	88.30%
*Body Mass Index (kg/m*^*)2*^						
< 25	156,241	33.40%	88	19.69%	724	27.88%
[25,30[	199,134	42.57%	186	41.61%	1058	40.74%
[30,40[	103,803	22.19%	149	33.33%	734	28.26%
> = 40	8,656	1.85%	24	5.37%	81	3.12%
**Biological**						
Cholesterol (mmol/L)	439,979	5.71 (1.13)	421	5.45 (1.26)	2446	5.48 (1.24)
HDL cholesterol (mmol/L)	402,733	1.45 (0.38)	392	1.34 (0.37)	2237	1.39 (0.40)
Triglycerides (mmol/L)	439,634	1.74 (1.02)	421	1.92 (1.08)	2445	1.84 (1.01)
Vitamin D (nmol/L)	420,153	48.78 (21.07)	398	46.03 (21.44)	2343	47.51 (21.91)
Cystatin C (mg/L)	439,942	0.90 (0.16)	421	1.01 (0.22)	2442	1.01 (0.27)
**Medical**						
*Cancer*						
No	399,570	84.93%	351	76.47%	1806	68.77%
Yes	70,895	15.07%	108	23.53%	820	31.23%
*Cardiovascular*						
No	381,505	81.09%	243	52.94%	1499	57.08%
Yes	88,960	18.91%	216	47.06%	1127	42.92%
*Hypertension*						
No	320,865	68.20%	176	38.34%	1223	46.57%
Yes	149,600	31.80%	283	61.66%	1403	53.43%
*Diabetes*						
No	437,687	93.03%	345	75.16%	2156	82.10%
Yes	32,778	6.97%	114	24.84%	470	17.90%
*Respiratory*						
No	374,220	79.54%	309	67.32%	1762	67.10%
Yes	96,245	20.46%	150	32.68%	864	32.90%
*Autoimmune*						
No	407,506	86.62%	357	77.78%	2092	79.66%
Yes	62,959	13.38%	102	22.22%	534	20.34%
*ACE inhibitors*						
No	427,168	90.80%	353	76.91%	2115	80.54%
Yes	43,297	9.20%	106	23.09%	511	19.46%
*Angiotensin II receptor blocker*						
No	453,016	96.29%	420	91.50%	2407	91.66%
Yes	17,449	3.71%	39	8.50%	219	8.34%
*Oral steroid*						
No	466,236	99.10%	442	96.30%	2571	97.91%
Yes	4229	0.90%	17	3.70%	55	2.09%
*Statin*						
No	398,609	84.73%	302	65.80%	1818	69.23%
Yes	71,856	15.27%	157	34.20%	808	30.77%
**Environmental**						
NO_X_ (ug/m^3^)	463,650	44.03 (15.50)	446	46.65 (15.38)	2590	45.10 (15.28)
PM10 (ug/m^3^)	432,557	16.23 (1.90)	403	16.50 (1.77)	2341	16.30 (1.87)
PM2.5 (absorbance/m)	432,557	1.19 (0.27)	403	1.22 (0.26)	2341	1.18 (0.26)
PM2.5 (ug/m^3^)	432,557	9.99 (1.06)	403	10.21 (1.02)	2341	10.06 (1.06)

### Multivariable analyses and variable selection

In the fully adjusted model (Supplementary Figure 3, Supplementary Table 4A), ORs for COVID-19 death were 2.76 [2.18–3.49] (*p* = 2.6 × 10^–17^) per standard deviation (8.1 years) for age, 1.47 [1.26–1.73] (*p* = 1.3 × 10^–6^) for male sex and 1.21 [1.12–1.29] (*p* = 3.0 × 10^–7^) for Black ethnicity. Most univariate associations were strongly attenuated when adjusted for age, sex, ethnicity and other covariates; 16 were associated with COVID-19 mortality when first included in sequential models. Associations for obesity and morbid obesity, and higher levels of cystatin C did not survive adjustment for biological or medical factors. In the fully adjusted model, in addition to age, male sex and Black ethnicity, COVID-19 mortality was associated with being a healthcare worker, current smoker, former drinker, cardiovascular disease, hypertension, diabetes, autoimmune disease and history of oral steroid use (Supplementary Figure 3, Supplementary Table 4A).

**Fig. 1:**
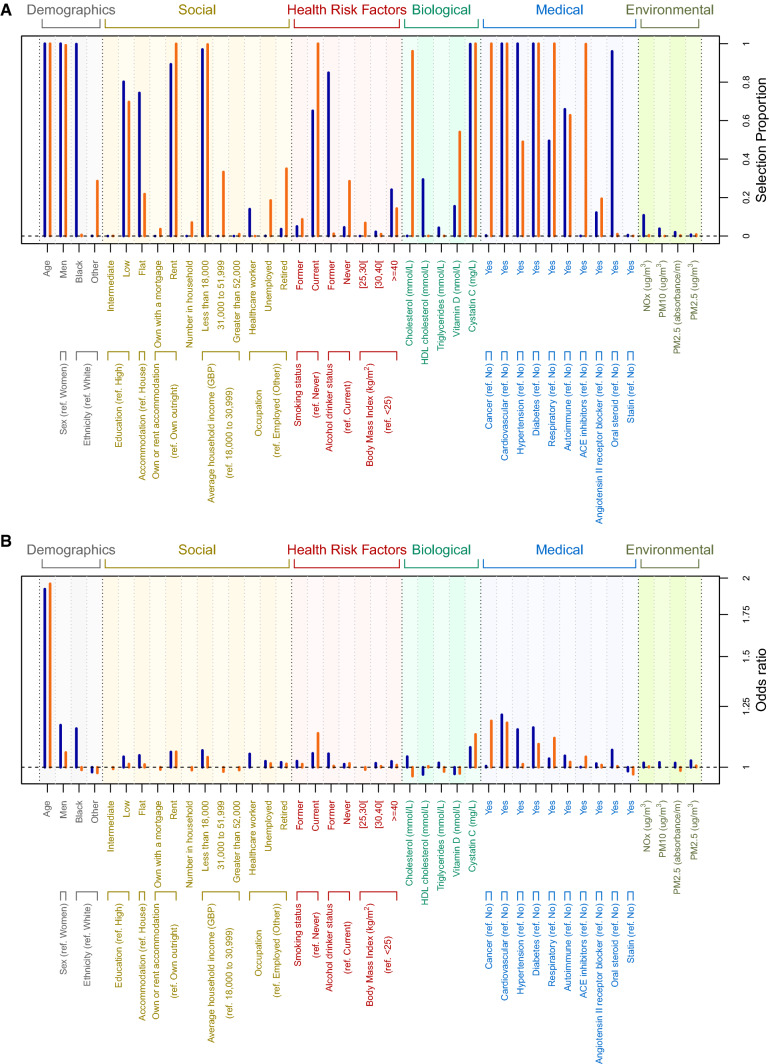
Selection proportion (**a**) and penalised odds ratios (**b**) from stability analyses based on logistic-LASSO models regressing jointly the demographic (in grey, N = 4), social (brown, N = 12), health risk (red, N = 7), biological (green, N = 6), medical (blue, N = 10), and environmental (olive green, N = 4) factors against the risk of COVID-19 death (in blue) and non-COVID-19 death (in orange). Selection proportion from stability analysis were inferred from 1000 models based on an 80% subsample of the population

Variable selection models consistently selected (≥ 96% selection proportion) age, male sex, Black ethnicity as well as earning less than GBP 18,000 per year, cystatin C, cardiovascular disease, hypertension, diabetes, and history of oral steroid use as jointly contributing to risk of COVID-19 death. Additionally, autoimmune and respiratory disease, social (low educational attainment, living in a flat, and renting), and health risk (current smokers and former drinkers) factors were highly selected (selection proportions ranging from 50 to 89%, Fig. 1a). Among selected variables, the strongest effects were for age, male sex, Black ethnicity, cardiovascular disease, hypertension and diabetes (Fig. 1b).

**Fig. 2:**
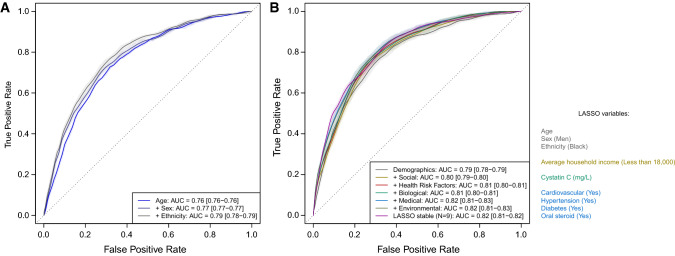
Receiver operating characteristic (ROC) curves from logistic regression models for risk of COVID-19 death. Results are presented for logistic models sequentially including age (light blue), sex (dark blue) and ethnicity (grey) (**a**). Results are also presented for a model sequentially including (N = 4) demographic (grey), (N = 12) social (beige), (N = 7) health risk (red), (N = 6) biological (green), (N = 10) medical (light blue), and (N = 4) environmental (olive green) factors, as well as a model including the (N = 7) factors consistently selected by logistic LASSO (selection proportion > 0.95) (purple) (**b**). Predictive performances were derived from a subsampling procedure (repeated independently 1000 times) of 80% of the study population as training set to produce ROC curves and corresponding AUC in the validation set (remaining 20%). The ROC curve and AUC point estimate corresponds to mean performance across 1000 subsamples, and the coloured areas (and AUC ranges) reflect the 1st and 99th percentiles of the performances yielded across the subsamples

ROC analyses showed that age alone was strongly explanatory of COVID-19 death with an average AUC of 0.76, increasing to 0.77 and 0.79 with sequential inclusion of sex and ethnicity, respectively (Fig. 2a). Both the saturated and LASSO models (Fig. 2b) yielded mean AUC of 0.82.

Analyses for non-COVID-19 mortality in the same period showed independent associations with age, male sex, renting, being unemployed, ever smoking, never drinking, cystatin C, history of taking ACEi, cancer, diabetes, and cardiovascular, autoimmune and respiratory diseases (Supplementary Figure 3, Supplementary Table 4B), and inversely with ethnicity other than Black or White, earning 31,000–51,999 GBP, cholesterol, vitamin D, and history of statin use. Penalised regression selected (selection proportion ≥ 96%) age, male sex, renting, earning less than 18,000 GBP, current smoking, cholesterol, cystatin C, history of taking ACEi, cancer, diabetes, and cardiovascular and respiratory disease as jointly contributing to non-COVID-19 mortality (Fig. 1a). Effect size estimates for age were much larger than all other covariates (Fig. 1b) and the LASSO model yielded an AUC of 0.77 (Supplementary Figure 4).

## Discussion

### Main findings

We found that age, male sex and Black ethnicity were strongly associated with COVID-19 death as previously reported [5, 6] and were highly explanatory of COVID-19 death. In addition, comorbidities (cardiovascular disease, hypertension, diabetes and autoimmune disease), history of oral steroids and being a healthcare worker, current smoker or former drinker at enrolment were independently associated with COVID-19 death. Age, male sex, Black ethnicity, cardiovascular disease, hypertension, diabetes, and history of oral steroid use were also highly selected in LASSO models, as were cystatin C and income. Of these, ethnicity, hypertension, and history of steroid use specifically associated with the risk of COVID-19 but not non-COVID-19 death in the same population and during the same period. These variables yielded only incremental improvements over age, sex and ethnicity in the prediction of COVID mortality.

We examined effects of various classes of drugs (steroids, RAAS inhibitors, statins) on risk of COVID-19 death. History of oral steroid use at enrolment was consistently associated with risk of COVID-19 death after multiple adjustment and in LASSO stability selection. These findings might result from the long-term immunosuppressant effects of systemic steroids or the associated risk of diabetes [36]; alternatively, they might be acting as a marker for severity of underlying disease such as autoimmune or respiratory disease. However, it has been shown that systemic steroids are an effective treatment for severe COVID-19, including reducing risk of COVID-19 mortality for those requiring oxygen therapy [37].

ACEi and ARBs have been postulated to increase risk of severe / fatal COVID-19 due to, among other possible mechanisms, upregulation of transmembrane ACE2 receptor expression (the cell entry site for the SARS-CoV-2 virus) [19]. In the present study, however, while history of ACEi and ARB use were positively associated with risk of COVID-19 death in univariate analysis, these associations did not survive multiple adjustment. This is in keeping with other reports showing no effect of these drugs on COVID-19 mortality [20, 21].

The role of statins in COVID-19 remains unclear. Positive effects have been proposed, for example through anti-inflammatory, anti-thrombotic or immunomodulatory mechanisms, as well as negative effects such as on kidney function or increased diabetes risk [24, 38, 39]. Here, statin therapy was positively associated with risk of COVID-19 death in univariate analysis but not after multiple adjustment, nor was it selected in LASSO stability analyses. It seems likely that the univariate association with statin therapy is confounded by comorbidities such as cardiovascular disease, where statins are used for prevention and treatment.

We found healthcare workers to be at increased risk of COVID-19 death even after adjustment for other covariates. These findings are consistent with results from national mortality statistics [40], which show elevated risk of COVID-19 mortality among healthcare workers (especially men) in comparison to that of the general population, accounting for age and sex. This may reflect a higher risk of infection among healthcare workers than in the general population [41].

A number of lifestyle and environmental factors have been suggested to affect risk of COVID-19 death. Among these, smoking has been suggested to reduce risk of infection but increase risk of severe or fatal COVID-19 post infection [15, 42]. In the present study, current smoking on enrolment was positively associated with risk of COVID-19 death. Meanwhile, respiratory disease was associated with COVID-19 mortality only in univariate analysis. The respiratory disease findings may partly be explained by inclusion of smoking in adjusted analyses. However, neither smoking nor respiratory disease were highly selected by LASSO models (< 50%), suggesting they were not key factors driving COVID-19 mortality despite SARS-CoV-2 virus being primarily a respiratory pathogen.

Environmental exposure to air pollutants [43] and low vitamin D levels have both been proposed to increase risk of COVID-19 death [16] but we found little support for these associations. While vitamin D was associated with decreased COVID-19 mortality risk in univariate analysis, this did not survive multiple adjustment nor was vitamin D selected by LASSO stability analysis; these findings are consistent with lack of association between vitamin D levels and positive testing for SARS-CoV-2 virus in previous analyses of UK Biobank [17]. For air pollutants, while we observed a small effect of particulate pollution on risk of COVID-19 death in univariate analyses, this was attenuated upon adjustment for other covariates.

Cystatin C was positively associated with COVID-19 mortality in univariate analysis and was highly selected by the LASSO models but did not survive multiple adjustment. Cystatin C has been implicated in severe COVID-19 [44] but, to our knowledge, this is the first report of it being associated with risk of COVID-19 death. It is a marker of kidney function and inflammatory state and may capture features of comorbidities, such as cardiovascular disease, that were independently associated with COVID-19 mortality in our data [45].

Our work has a number of limitations. First, although UK Biobank includes over 500,000 participants, numbers of COVID-19 deaths were modest compared to national studies of mortality and hospitalised cases. Nonetheless, unlike such studies, our work combines (i) COVID-19 and non-COVID-19 mortality data linked to UK Biobank data, (ii) individual demographic, social, biological, health risk, medical and environmental factors collected at enrolment, and (iii) detailed information on premorbid conditions. While baseline characteristics of the cohort were obtained over ten years prior to the period of the epidemic, they may have changed in the interim. However, for the intervening period, we were able to identify morbid events through linkage to hospitalisation data, giving updated information on comorbidities. UK Biobank has a 5.5% response rate, giving a selected population that is not fully representative of the UK population [46]. However, it has been reported that within-cohort risk factor associations with mortality in UK Biobank appear generalisable. Data from the latest release of UK Biobank include COVID-19 deaths up to the end of September 2020, and therefore do not capture the second wave of the epidemic in the UK. Given the bimodal nature of the pattern of COVID-19 mortality in the UK so far, timing of the occurrence of COVID-19 deaths will need to be taken into account in future analyses, for example, using survival regression models.

The use of multivariable regression and variable selection approaches enabled us to model correlation across predictors in relation to mortality and identify sets of variables jointly contributing to risk of COVID-19 death. These methods aim to capture the complex interrelationships between covariates, although are dependent on parametric assumptions underlying (generalised) linear models. In addition, given these are observational data, we cannot rule out residual confounding. However, comparing our findings for COVID-19 versus non-COVID-19 mortality during the same period lends further plausibility to the specificity of the COVID-19 mortality associations.

In conclusion, our study of the ongoing COVID-19 epidemic as it affected UK Biobank participants has identified age, male sex and Black ethnicity as key explanatory factors for COVID-19 death. Among other covariates, some were consistently associated with and moderately explanatory of COVID-19 mortality. Comorbidities including cardiovascular disease, hypertension, diabetes and autoimmune disease as well as oral steroid use at enrolment were independently associated with increased COVID-19 mortality risk. In particular, Black ethnicity, oral steroids and hypertension were associated with COVID-19 but did not explain non-COVID-19 mortality in this population. Our results indicate that previously reported associations with COVID-19 mortality involving the use of RAAS inhibitors, statins, current smoking, vitamin D levels and air pollutants may, at least partially, be explained by factors we have identified. Further follow-up of UK Biobank with linkage to primary and secondary care as well as future mortality data will help delineate the long-term sequelae of COVID-19.

## Supplementary Information

Below is the link to the electronic supplementary material.

Supplementary file1 (PDF 791 KB)

## Data Availability

No additional data available.
