# Supplementary material for: COVID-19 mortality in the UK Biobank cohort: revisiting and evaluating risk factors
Source: Eur J Epidemiol. 2021 Feb 15;36(3):299–309. doi: 10.1007/s10654-021-00722-y (PMC7882869; doi:10.1007/s10654-021-00722-y)
Supplement: Supplementary file 1 — Supplementary file1 (PDF 791 KB) [file 10654_2021_722_MOESM1_ESM.pdf]

Supplementary Information
COVID-19 mortality in the UK Biobank cohort: revisiting and evaluating risk factors

Dr Joshua Elliott[a,b,c,†]; Ms Barbara Bodinier [a,b,†]; Mr Matthew Whitaker[a,b]; Dr Cyrille Delpierre [d]; Prof. Ioanna Tzoulaki[a,b,e]; Prof. Roel Vermeulen[f], Prof. Paul Elliott[a,b,‡] ; Prof. Marc Chadeau-Hyam[a,b, ‡]

[a] Department of Epidemiology and Biostatistics, School of Public Health, Imperial College London, London, UK

[b] MRC Centre for Environment and Health, Imperial College, London, UK

[c] Royal Surrey County Hospital, Guildford, Surrey, GU2 7XX

[d] UMR LEASP, Université de Toulouse III, UPS, Inserm, Toulouse, France

[e] Department of Hygiene and Epidemiology, University of Ioannina Medical School, Ioannina, Greece

[f] Institute for Risk Assessment Sciences (IRAS), Utrecht University, Utrecht, The Netherlands

[†] Joint first authors

[‡] Joint last authors

Variable definition

Demographics

Age was calculated using the year and month of birth (UK Biobank fields 34 and 52) as of 31 January 2020, when COVID-19 was first diagnosed in the UK. Sex is as defined in field 31. Ethnicity was recoded in three categories from field 21000: White (British, Irish or any other White background), Black (including Caribbean and African background) and Other (Mixed, Asian or other ethnic background).

Social factors

Highest attained qualification is retrieved from field 6138. The housing variables are encoded in fields 670, 680, and 709. The household income (field 738) is recoded in four balanced categories. Occupation is defined from the job code based on the SOC2000 classification (field 132) and the employment status (field 6142) entered at baseline, and recoded as: employed (in paid employment or self-employed) healthcare workers (including health professionals, health and social welfare associate professionals, healthcare and related personal services, health and social service managers and hospital porters), employed non-healthcare workers, unemployed (including studying and doing unpaid voluntary work), and retired.

Health risk factors

Smoking and alcohol drinking status are used as defined in fields 20116 and 20117 respectively. Continuous measurement of the Body Mass Index (BMI) at recruitment is provided in field 21001 and was categorised as: <25, 25–30, 30–40 and >40 kg/m^2.

Biological variables

Measured levels of total cholesterol in mmol/L (as defined in UK Biobank field ID 30690), high density lipoprotein cholesterol in mmol/L (30760), triglycerides in mmol/L (30870), vitamin D in nmol/L (30890), and cystatin C in mg/L (30720) were taken at baseline.

Medical variables

All codes used to define medical variables are available in Supplementary Table 1. Comorbidities (yes/no) were identified either from nurse-administrated questionnaire data at baseline (UK Biobank fields 20001 and 20002) or linked Hospital Episode Statistics (ICD-9 or ICD-10 codes, censored at 31 March 2017). Cancer included any reported or diagnosed non-benign cancer. Diabetes additionally included those with baseline glycated haemoglobin above or equal to 48 mmol/mol. Medication codes were from self-report (UK Biobank field 20003).

Environmental exposures

Modelled levels of exposures to NO_X, PM10, PM2.5 absorbance and PM2.5 were used as defined in fields 24004, 24005, 24007, and 24006.

Supplementary Figure 1: Results from the univariate logistic regression models from each covariate separately for the risk of COVID-19 death (in blue) and non-COVID-19 death (in orange). Effect size estimates expressed as odds ratios (ORs) are represented for demographic covariates, and social, health risk, biological, medical, and environmental factors. ORs for continuous variables are standardized to ensure comparability.

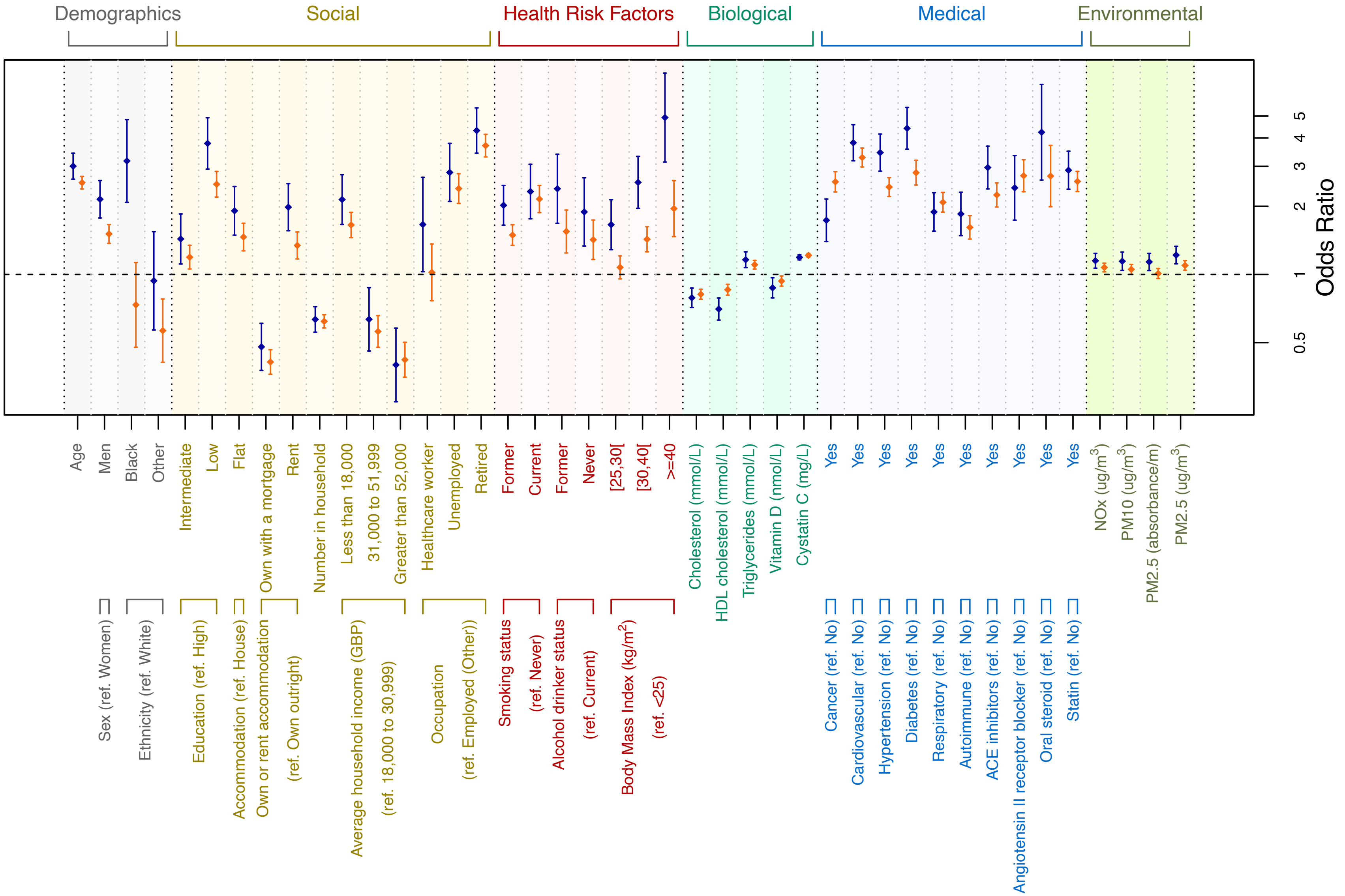

Supplementary Figure 2: Comparison of the odds ratio (A) and p-values (B) from the logistic models of the probability of dying from COVID-19, where the cause of death is defined as U07.1 and U07.2 (X-axis, 459 deaths vs. 470,465 participants alive at the end of follow up), or U07.1 only (Y-axis, 438 deaths vs. 470,465 participants alive at the end of follow up) for demographics (in grey, N=4), social (brown, N=12), health risk (red, N=7), biological (green, N=5), medical (blue, N=10), and environmental (olive green, N=4) factors.

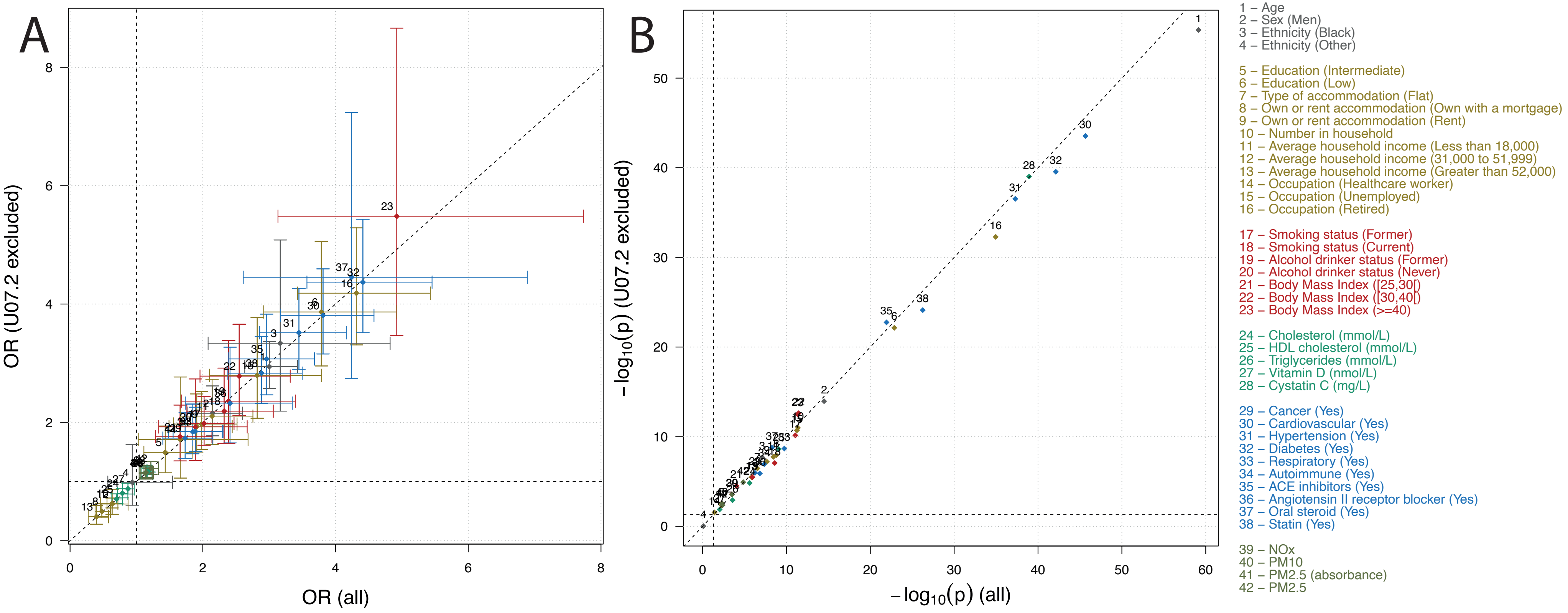

Supplementary Figure 3. Odds ratio [95% confidence intervals] from logistic models for risk of COVID-19 death (in blue) and non-COVID-19 death during the same period (in orange). Results are presented for sequentially adjusted models: demographic (Model 1), with additional adjustment for social (Model 2), health risk (Model 3), biological (Model 4), medical (Model 5), and environmental factors (Model 6).

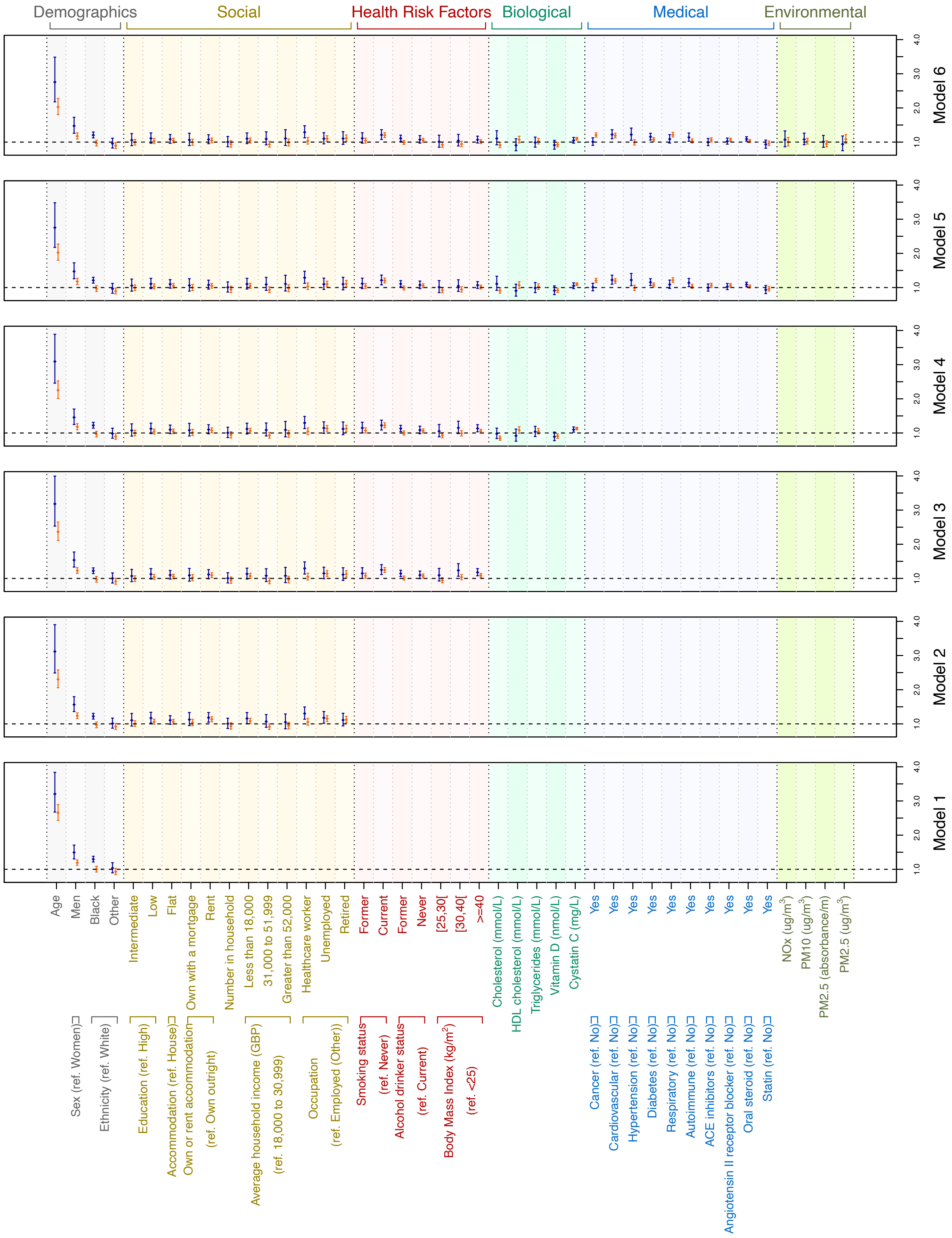

Supplementary Figure 4: Receiver operating characteristic (ROC) curves from logistic regression models for the risk of non-COVID-19 death between 31 January and 21 September 2020. ROC curves are presented for a logistic model including (N=4) demographics covariates (grey) and the (N=12) factors consistently selected by the logistic LASSO (purple). Predictive performances were derived from a subsampling procedure (repeated independently 1,000 times) of 80% of the study population as training set to produce ROC curves and corresponding AUC in the validation set (remaining 20%). The ROC curve and AUC point estimate corresponds to mean performance across the 1,000 subsamples, and the coloured areas (and AUC ranges) reflect the 1st and 99th percentiles of the performances yielded across the subsamples.

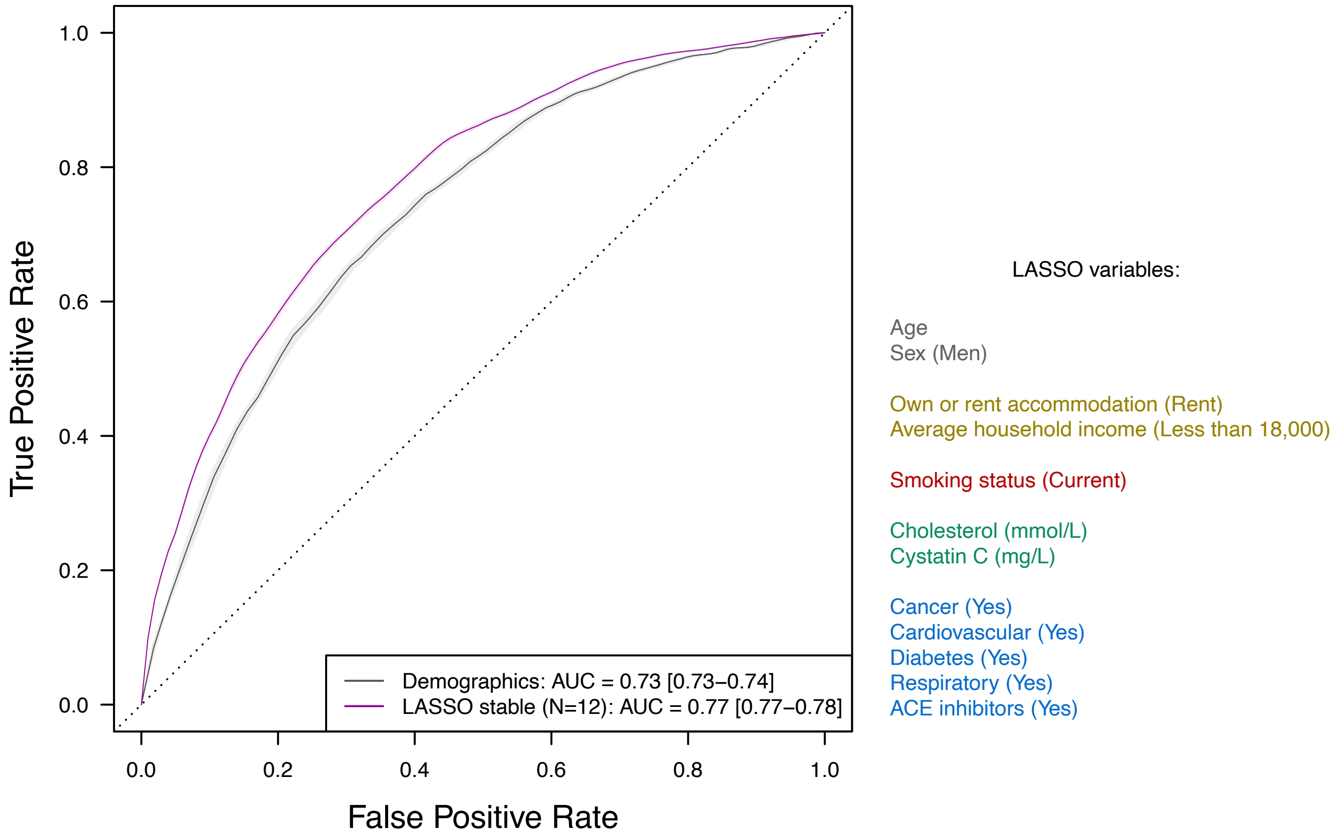

Supplementary Table 1. Codes for (A) comorbidities using UK Biobank data coding 3 and 6 (self-report, UK Biobank fields 20001 and 20002) and linkage to Hospital Episode Statistics (ICD-9 and ICD-10) and (B) medication use at baseline (data coding 4, UK Biobank field 20003).

A

Cancer			Cardiovascular			Hypertension			Diabetes			Respiratory			Autoimmune (1/2)			Autoimmune (2/2)		
Field 20001	ICD-10	ICD-9	Field 20002	ICD-10	ICD-9	Field 20002	ICD-10	ICD-9	Field 20002	ICD-10	ICD-9	Field 20002	ICD-10	ICD-9	Field 20002	ICD-10	ICD-9	Field 20002	ICD-10	ICD-9
Any	C00-C09	140-149	1066	I00-I02	390-392	1065	I10-I15	401-405	1220	E10	250	1111	J30-J39	470-478	1224	D68.3	286.5	1435	L40	710
	C15-C26	150-159	1067	I05-I09	393-398	1072			1222	E11		1112	J40-J47	480-488	1225	D68.4	286.7	1453	L43	720.0
	C30-C39	160-165	1068	I20-I25	410-414				1223	E12		1113	J60-J70	490-496	1226	D68.5	289.81	1461	L63.9	720.8
	C40-C41	170-176	1074	I26-I28	415-417					E13		1114	J80-J84	500-508	1228	D69.3	287.31	1462	L80	281.0
	C43-C44	179-189	1075	I30-I52	420-429					E14		1115	J85-J86	510-519	1234	D86	135	1463	L90.0	
	C45-C49	190-199	1076	I60-I69	430-438							1117	J90-J94		1256	D89	245.2	1464	M05	
	C50	200-209	1077	I70-I76	440-445							1120	J95-J99		1260	E06.3	340	1475	M06	
	C51-C58	230-234	1078	I77.0-I77.5	447.0-447.5							1121			1261	E27.1	357.81	1477	M07	
	C60-C63	235-238	1079	I77.7-I77.9	447.7-447.9							1122			1313	E27.2	358	1480	M08	
	C64-C68	239	1080	I78-I79	448-449							1123			1331	G35	377.3	1481	M30	
	C69-C72		1081	I81-I82	453							1124			1345	G61	447.6	1506	M31	
	C73-C75		1082	I95-I99								1125			1371	G70	555	1520	M32	
	C76-C80		1083									1126			1372	H46	556	1522	M33	
	C82-C96		1086												1376	I77.6	571.6	1549	M34	
	C97		1087												1377	K50	571.42	1550	M35	
	D00-D09		1088												1378	K51	576.1	1561	M45	
	D37-D48		1093												1379	K74.3	579.0	1564	M46.0	
			1094												1380	K75.4	694	1609	M46.1	
															1381	K83.01	696	1661	M46.2	
															1382	K90.0	697.0	1667	M46.4	
															1383	L10	704.01		M46.8	
															1384	L11	709.01		M46.9	
															1397	L12	701.0		N08	
															1428	L13	714		D51.0	
															1426	L14	446			

B

Angiotensin-converting enzyme inhibitors		Angiotensin II receptor blockers		Oral steroids		Statins	
Code	Medication	Code	Medication	Code	Medication	Code	Medication
1140860728	Quinapril	1140916356	losartan	1140868364	prednisone	1141192410	rosuvastatin
1140860738	quinalapril+hydrochlorothiazide 10mg/12.5mg tablet	1140916362	cozaar half strength 25mg tablet (losartan)	1140874930	prednisolone	1140861958	simvastatin
1140860752	acepril 12.5mg tablet (captopril)	1141145660	valsartan	1140874896	hydrocortisone	1140861970	lipostat 10mg tablet
1140860758	capoten 12.5mg tablet (captopril)	1141151016	losartan potassium+hydrochlorothiazide 50mg/12.5mg tablet	1140874936	deltacortril enteric 2.5mg e/c tablet	1140888648	pravastatin
1140860764	captopril+hydrochlorothiazide 25mg/12.5mg tablet	1141151018	cozaar-comp 50mg/12.5mg tablet (losartan)	1140874940	deltastab 1mg tablet	1140910654	velastatin
1140860776	innovace 2.5mg tablet (enalapril)	1141152998	irbesartan	1140874944	precortisyl 1mg tablet	1140910632	eptastatin
1140860784	innozide tablet (enalapril + HCTZ)	1141153006	aprovel 75mg tablet (irbesartan)	1140874950	prednesol 5mg tablet	1141146234	atorvastatin
1140860790	enalapril maleate+hydrochlorothiazide 20mg/12.5mg tablet	1141156836	candesartan cilexetil	1140874954	hydrocortistab 20mg tablet	1141146138	lipitor 10mg tablet
1140860802	coversyl 2mg tablet (perindopril)	1141156846	amias 2mg tablet	1140874956	hydrocortone 10mg tablet	1140864592	lescol 20mg capsule
1140860806	ramipril	1141166006	telmisartan	1140874976	methylprednisolone	1141192414	crestor 10mg tablet
1140860736	accuretic tablet (quinapril + HCTZ)	1141171336	eprosartan	1140874978	medrone 2mg tablet	1141200040	zocor heart-pro 10mg tablet
1140860738	quinalapril+hydrochlorothiazide 10mg/12.5mg tablet	1141171344	teveten 300mg tablet (eprosartan)	1141157402	prednisolone product	1140881748	zocor 10mg tablet
1140860878	staril 10mg tablet (fosinopril)	1141172682	irbesartan+hydrochlorothiazide 150mg/12.5mg tablet	1140910634	deltahydrocortisone		
1140860882	cilazapril	1141172686	coaprovel 150mg/12.5mg tablet	1141145782	deflazacort		
1140860892	vascace 250micrograms tablet (cilazapril)	1141187788	telmisartan+hydrochlorothiazide 40mg/12.5mg tablet	1140874790	betamethasone		
1140860904	trandolapril	1141193282	olmesartan	1140874816	dexamethasone		
1140860912	gopten 500micrograms capsule (trandolapril)	1141201038	valsartan+hydrochlorothiazide 80mg/12.5mg tablet				
1140860918	odrik 500micrograms capsule (trandolapril)	1141201040	co-diovan 80mg/12.5mg tablet (valsartan + HCTZ)				
1140860938	deponit 5mg/24hours transdermal patch						
1140864952	lisinopril+hydrochlorothiazide 10mg/12.5mg tablet						
1140888552	enalapril						
1140888556	fosinopril						
1140888560	perindopril						
1140923712	moexipril						
1141151382	hypapril 12.5mg tablet (captopril)						
1141153328	trandolapril+verapamil hydrochloride						
1141164148	imidapril hydrochloride						
1141165470	felodipine+ramipril						
1141167822	tensopril 12.5mg tablet (captorpil)						
1141180592	perindopril+indapamide						
1140860696	lisinopril						
1140860706	carace 2.5mg tablet (lisinopril + HCTZ)						
1140860714	zestril 2.5mg tablet (lisinopril)						

Supplementary Table 2. Results from univariate logistic models for risk of COVID-19 and non-COVID-19 death in the full UK Biobank sample. Effect size estimates are expressed as odds ratios [95% confidence intervals] with p-values.

			COVID-19 deaths		Non-COVID-19 deaths	
			OR	p-value	OR	p-value
**Demographics**	Age (years)		3.00 [2.63-3.43]	$7.24x10^{-60}$	2.55 [2.42-2.68]	$1.98x10^{-275}$
	Sex (ref. Women)	*Men*	2.15 [1.78-2.60]	$3.25x10^{-15}$	1.57 [1.45-1.70]	$2.00x10^{-30}$
	Ethnicity (ref. White)	*Black*	3.17 [2.08-4.82]	$7.69x10^{-8}$	0.61 [0.42-0.89]	$1.08x10^{-2}$
		Other	0.94 [0.57-1.55]	$8.00x10^{-1}$	0.56 [0.43-0.72]	$1.14x10^{-5}$
**Social**	Education (ref. High)	*Intermediate*	1.43 [1.11-1.85]	$5.41x10^{-3}$	1.22 [1.10-1.34]	$7.42x10^{-5}$
		Low	3.79 [2.92-4.91]	$1.37x10^{-23}$	2.48 [2.23-2.76]	$3.51x10^{-64}$
	Type of accommodation (ref. House)	*Flat*	1.91 [1.49-2.44]	$2.93x10^{-7}$	1.40 [1.25-1.57]	$9.55x10^{-9}$
	Own/Rent (ref. Own outright)	*Own with a mortgage*	0.48 [0.38-0.61]	$1.62x10^{-9}$	0.41 [0.37-0.46]	$2.31x10^{-67}$
		Rent	1.98 [1.56-2.52]	$2.13x10^{-8}$	1.29 [1.15-1.44]	$8.56x10^{-6}$
	Number in household		0.63 [0.56-0.72]	$4.29x10^{-12}$	0.63 [0.60-0.66]	$2.21x10^{-64}$
	Income (GBP) (ref. 18,000 to 30,999)	*Less than 18,000*	2.14 [1.66-2.76]	$3.61x10^{-9}$	1.54 [1.39-1.71]	$2.36x10^{-16}$
		31,000 to 51,999	0.63 [0.46-0.87]	$5.47x10^{-3}$	0.57 [0.50-0.65]	$3.85x10^{-18}$
		Greater than 52,000	0.40 [0.27-0.58]	$1.46x10^{-6}$	0.43 [0.37-0.49]	$7.73x10^{-33}$
	Occupation (ref. Employed (Other))	*Healthcare worker*	1.66 [1.03-2.68]	$3.81x10^{-2}$	1.02 [0.81-1.28]	$8.60x10^{-1}$
		Unemployed	2.82 [2.10-3.79]	$5.61x10^{-12}$	2.24 [1.98-2.53]	$7.64x10^{-39}$
		Retired	4.31 [3.43-5.43]	$1.12x10^{-35}$	3.48 [3.18-3.81]	$2.26x10^{-158}$
**Health Risk Factors**	Smoking status (ref. Never)	*Former*	2.02 [1.65-2.47]	$8.69x10^{-12}$	1.55 [1.42-1.69]	$3.18x10^{-24}$
		Current	2.32 [1.76-3.06]	$2.50x10^{-9}$	2.21 [1.98-2.47]	$6.95x10^{-45}$
	Alcohol drinker status (ref. Current)	*Former*	2.39 [1.68-3.39]	$1.16x10^{-6}$	1.62 [1.36-1.93]	$4.06x10^{-8}$
		Never	1.89 [1.34-2.67]	$3.22x10^{-4}$	1.51 [1.29-1.77]	$2.26x10^{-7}$
	BMI (kg/m^2) (ref. <25)	*[25,30[*	1.66 [1.29-2.14]	$9.30x10^{-5}$	1.15 [1.04-1.26]	$4.68x10^{-3}$
		[30,40[	2.55 [1.96-3.32]	$3.52x10^{-12}$	1.53 [1.38-1.69]	$8.62x10^{-16}$
		>=40	4.92 [3.13-7.73]	$4.73x10^{-12}$	2.02 [1.60-2.54]	$2.34x10^{-9}$
**Biological**	Cholesterol (mmol/L)		0.79 [0.71-0.87]	$2.43x10^{-6}$	0.82 [0.78-0.85]	$1.34x10^{-22}$
	HDL cholesterol (mmol/L)		0.70 [0.63-0.79]	$8.36x10^{-10}$	0.84 [0.80-0.88]	$5.76x10^{-15}$
	Triglycerides (mmol/L)		1.16 [1.07-1.26]	$2.71x10^{-4}$	1.10 [1.06-1.14]	$3.93x10^{-7}$
	Vitamin D (nmol/L)		0.87 [0.79-0.97]	$9.25x10^{-3}$	0.94 [0.90-0.98]	$3.57x10^{-3}$
	Cystatin C (mg/L)		1.19 [1.16-1.22]	$1.18x10^{-39}$	1.25 [1.23-1.28]	$2.01x10^{-111}$
**Medical**	Cancer (ref. No)	*Yes*	1.73 [1.40-2.15]	$5.73x10^{-7}$	2.56 [2.36-2.78]	$2.75x10^{-109}$
	Cardiovascular (ref. No)	*Yes*	3.81 [3.17-4.58]	$2.24x10^{-46}$	3.22 [2.98-3.48]	$4.61x10^{-192}$
	Hypertension (ref. No)	*Yes*	3.45 [2.86-4.16]	$5.15x10^{-38}$	2.46 [2.28-2.66]	$1.78x10^{-116}$
	Diabetes (ref. No)	*Yes*	4.41 [3.57-5.45]	$7.56x10^{-43}$	2.91 [2.63-3.22]	$1.31x10^{-96}$
	Respiratory (ref. No)	*Yes*	1.89 [1.55-2.29]	$1.78x10^{-10}$	1.91 [1.76-2.07]	$4.80x10^{-54}$
	Autoimmune (ref. No)	*Yes*	1.85 [1.48-2.30]	$4.44x10^{-8}$	1.65 [1.50-1.82]	$5.98x10^{-25}$
	ACE inhibitors	*Yes*	2.96 [2.38-3.68]	$1.17x10^{-22}$	2.38 [2.16-2.63]	$8.33x10^{-69}$
	Angiotensin II receptor blocker	*Yes*	2.41 [1.74-3.35]	$1.51x10^{-7}$	2.36 [2.06-2.71]	$9.75x10^{-34}$
	Oral steroid	*Yes*	4.24 [2.61-6.89]	$5.42x10^{-9}$	2.36 [1.80-3.09]	$3.95x10^{-10}$
	Statin	*Yes*	2.88 [2.38-3.50]	$5.58x10^{-27}$	2.47 [2.27-2.68]	$3.65x10^{-100}$
**Environmental**	NO_X (ug/m^3)		1.15 [1.07-1.24]	$2.98x10^{-4}$	1.07 [1.03-1.10]	$4.74x10^{-4}$
	PM10 (ug/m^3)		1.14 [1.04-1.26]	$4.77x10^{-3}$	1.03 [0.99-1.08]	$1.16x10^{-1}$
	PM2.5 (absorbance/m)		1.14 [1.04-1.24]	$4.29x10^{-3}$	1.00 [0.96-1.04]	$8.42x10^{-1}$
	PM2.5 (ug/m^3)		1.22 [1.11-1.33]	$1.43x10^{-5}$	1.07 [1.03-1.11]	$7.76x10^{-4}$

Supplementary Table 3: Characteristics of the (N=459) COVID-19 deaths in the UK Biobank study. Results are presented for both COVID-19 ICD codes: U07.1 – COVID-19 death with virus identified, and U07.2 probable COVID-19 death (without identification of the virus).

		U071 (N=438)		U072 (N=21)	
		N	Mean (sd) / Proportion	N	Mean (sd) / Proportion
**Demographics**	Age (years)	438	74.42 (5.97)	21	76.19 (5.48)
	Sex				
	Women	160	36.53%	8	38.10%
	Men	278	63.47%	13	61.90%
	Ethnicity				
	White	394	90.99%	21	100.00%
	Black	23	5.31%	0	0.00%
	Other	16	3.70%	0	0.00%
**Social**	Education				
	High	81	19.42%	6	30.00%
	Intermediate	182	43.65%	6	30.00%
	Low	154	36.93%	8	40.00%
	Type of accommodation				
	House	351	82.78%	18	85.71%
	Flat	73	17.22%	3	14.29%
	Own or rent accommodation				
	Own outright	245	58.33%	13	65.00%
	Own with a mortgage	89	21.19%	2	10.00%
	Rent	86	20.48%	5	25.00%
	Number in household	423	2.06 (1.04)	20	2.10 (0.97)
	Average household income (GBP)				
	Less than 18,000	161	46.53%	10	52.63%
	18,000 to 30,999	89	25.72%	4	21.05%
	31,000 to 51,999	58	16.76%	4	21.05%
	Greater than 52,000	38	10.98%	1	5.26%
	Occupation				
	Unemployed	75	17.32%	3	14.29%
	Employed (Healthcare worker)	20	4.62%	0	0.00%
	Employed (Other)	99	22.86%	3	14.29%
	Retired	239	55.20%	15	71.43%
**Health Risk Factors**	Smoking status				
	Never	166	38.43%	5	23.81%
	Former	201	46.53%	10	47.62%
	Current	65	15.05%	6	28.57%
	Alcohol drinker status				
	Never	34	7.83%	1	4.76%
	Former	32	7.37%	2	9.52%
	Current	368	84.79%	18	85.71%
	Body Mass Index (kg/m^2)				
	<25	79	18.54%	9	42.86%
	[25,30[	177	41.55%	9	42.86%
	[30,40[	146	34.27%	3	14.29%
	>=40	24	5.63%	0	0.00%
**Biological**	Cholesterol (mmol/L)	403	5.46 (1.26)	18	5.10 (1.24)
	HDL cholesterol (mmol/L)	375	1.34 (0.36)	17	1.32 (0.43)
	Triglycerides (mmol/L)	403	1.90 (1.08)	18	2.29 (1.02)
	Vitamin D (nmol/L)	380	46.10 (21.54)	18	44.53 (19.68)
	Cystatin C (mg/L)	403	1.02 (0.22)	18	0.94 (0.20)
**Medical**	Cancer				
	No	335	76.48%	16	76.19%
	Yes	103	23.52%	5	23.81%
	Cardiovascular				
	No	232	52.97%	11	52.38%
	Yes	206	47.03%	10	47.62%
	Hypertension				
	No	166	37.90%	10	47.62%
	Yes	272	62.10%	11	52.38%
	Diabetes				
	No	330	75.34%	15	71.43%
	Yes	108	24.66%	6	28.57%
	Respiratory				
	No	297	67.81%	12	57.14%
	Yes	141	32.19%	9	42.86%
	Autoimmune				
	No	341	77.85%	16	76.19%
	Yes	97	22.15%	5	23.81%
	ACE inhibitors				
	No	334	76.26%	19	90.48%
	Yes	104	23.74%	2	9.52%
	Angiotensin II receptor blocker				
	No	402	91.78%	18	85.71%
	Yes	36	8.22%	3	14.29%
	Oral steroid				
	No	421	96.12%	21	100.00%
	Yes	17	3.88%	0	0.00%
	Statin				
	No	290	66.21%	12	57.14%
	Yes	148	33.79%	9	42.86%
**Enviro**	NO_X (ug/m^3)	426	46.72 (15.42)	20	45.14 (14.64)
	PM10 (ug/m^3)	390	16.52 (1.76)	13	15.85 (2.07)
	PM2.5 (absorbance/m)	390	1.23 (0.26)	13	1.21 (0.20)
	PM2.5 (ug/m^3)	390	10.22 (1.01)	13	10.04 (1.24)

Supplementary Table 4. Odds ratio [95% confidence intervals], and p-values from logistic models for the probability of COVID-19 death (A) and non-COVID-19 death (B) in the full UK Biobank sample. Results are represented for sequentially adjusted models, where benchmark predictors are defined as demographic descriptors (D, Model 1), and models are additionally adjusted for social (S, Model 2), health risk (HRF, Model 3), biological (B, Model 4), medical (M, Model 5), and environmental factors (E, Model 6).

			Model 1		Model 2		Model 3		Model 4		Model 5		Model 6	
			OR	p-value	OR	p-value	OR	p-value	OR	p-value	OR	p-value	OR	p-value
D	Age (years)		3.20 [2.68-3.84]	9.78x10^{-37}	3.12 [2.49-3.90]	3.53x10^{-23}	3.18 [2.53-3.99]	2.39x10^{-23}	3.09 [2.46-3.89]	4.63x10^{-22}	2.75 [2.18-3.48]	2.75x10^{-17}	2.76 [2.18-3.49]	2.61x10^{-17}
	Sex (ref. Women)	*Men*	1.49 [1.30-1.71]	6.63x10^{-9}	1.56 [1.36-1.80]	2.67x10^{-10}	1.54 [1.33-1.77]	3.05x10^{-9}	1.46 [1.25-1.70]	2.09x10^{-6}	1.47 [1.26-1.72]	1.43x10^{-6}	1.47 [1.26-1.73]	1.30x10^{-6}
	Ethnicity (ref. White)	*Black*	1.30 [1.22-1.38]	1.82x10^{-15}	1.22 [1.14-1.31]	6.20x10^{-9}	1.22 [1.14-1.31]	8.67x10^{-9}	1.22 [1.14-1.31]	1.45x10^{-8}	1.21 [1.13-1.30]	8.90x10^{-8}	1.21 [1.12-1.29]	3.00x10^{-7}
		Other	1.03 [0.90-1.19]	6.46x10^{-1}	1.01 [0.87-1.17]	8.82x10^{-1}	1.00 [0.87-1.16]	9.75x10^{-1}	0.98 [0.85-1.14]	8.25x10^{-1}	0.97 [0.83-1.12]	6.46x10^{-1}	0.96 [0.83-1.11]	5.82x10^{-1}
S	Education	*Intermediate*			1.11 [0.94-1.30]	2.34x10^{-1}	1.07 [0.91-1.26]	4.22x10^{-1}	1.07 [0.91-1.27]	4.03x10^{-1}	1.06 [0.89-1.25]	5.22x10^{-1}	1.06 [0.90-1.25]	5.10x10^{-1}
	(ref. High)	*Low*			1.17 [1.02-1.34]	2.13x10^{-2}	1.12 [0.98-1.29]	8.69x10^{-2}	1.13 [0.98-1.29]	8.70x10^{-2}	1.11 [0.97-1.27]	1.35x10^{-1}	1.11 [0.97-1.27]	1.34x10^{-1}
	Accomm. (ref. House)	*Flat*			1.11 [0.99-1.24]	8.07x10^{-2}	1.10 [0.98-1.23]	9.83x10^{-2}	1.10 [0.98-1.23]	1.03x10^{-1}	1.10 [0.98-1.23]	9.85x10^{-2}	1.09 [0.97-1.22]	1.68x10^{-1}
	Own	*Own with a mortgage*			1.13 [0.95-1.33]	1.73x10^{-1}	1.09 [0.92-1.29]	3.16x10^{-1}	1.08 [0.91-1.28]	3.67x10^{-1}	1.06 [0.90-1.26]	4.86x10^{-1}	1.06 [0.90-1.26]	4.81x10^{-1}
	(ref. Own outright)	*Rent*			1.18 [1.05-1.33]	5.74x10^{-3}	1.12 [0.99-1.26]	7.35x10^{-2}	1.10 [0.98-1.24]	1.15x10^{-1}	1.08 [0.95-1.21]	2.29x10^{-1}	1.08 [0.95-1.21]	2.27x10^{-1}
	Number in household				1.00 [0.86-1.16]	9.62x10^{-1}	1.01 [0.87-1.17]	9.37x10^{-1}	1.01 [0.87-1.17]	9.44x10^{-1}	1.01 [0.87-1.16]	9.41x10^{-1}	1.00 [0.86-1.16]	9.66x10^{-1}
	Income (GBP)	*<18,000*			1.16 [1.01-1.33]	3.95x10^{-2}	1.13 [0.98-1.30]	8.47x10^{-2}	1.12 [0.97-1.29]	1.19x10^{-1}	1.10 [0.96-1.27]	1.73x10^{-1}	1.10 [0.96-1.27]	1.78x10^{-1}
	(ref. 18,000	*31,000-51,999*			1.07 [0.90-1.27]	4.47x10^{-1}	1.08 [0.91-1.28]	3.86x10^{-1}	1.09 [0.91-1.29]	3.48x10^{-1}	1.09 [0.92-1.30]	3.23x10^{-1}	1.09 [0.92-1.30]	3.13x10^{-1}
	to 30,999)	*>52,000*			1.05 [0.86-1.29]	6.40x10^{-1}	1.08 [0.88-1.32]	4.73x10^{-1}	1.09 [0.89-1.34]	4.11x10^{-1}	1.11 [0.90-1.36]	3.38x10^{-1}	1.11 [0.90-1.36]	3.29x10^{-1}
	Occup.	*Healthcare worker*			1.30 [1.14-1.49]	1.53x10^{-4}	1.29 [1.13-1.49]	2.26x10^{-4}	1.29 [1.13-1.48]	2.39x10^{-4}	1.29 [1.12-1.48]	3.02x10^{-4}	1.29 [1.12-1.48]	2.88x10^{-4}
	(ref. Employed	*Unemployed*			1.18 [1.02-1.36]	2.76x10^{-2}	1.15 [0.99-1.33]	6.39x10^{-2}	1.15 [0.99-1.33]	6.98x10^{-2}	1.10 [0.95-1.27]	2.14x10^{-1}	1.10 [0.95-1.28]	2.05x10^{-1}
	(Other))	*Retired*			1.11 [0.94-1.31]	2.11x10^{-1}	1.11 [0.94-1.31]	2.07x10^{-1}	1.12 [0.95-1.32]	1.81x10^{-1}	1.10 [0.93-1.30]	2.59x10^{-1}	1.10 [0.93-1.30]	2.43x10^{-1}
HRF	Smoking status	*Former*					1.15 [1.00-1.31]	4.58x10^{-2}	1.15 [1.00-1.31]	4.55x10^{-2}	1.11 [0.97-1.27]	1.24x10^{-1}	1.11 [0.97-1.27]	1.29x10^{-1}
	(ref. Never)	*Current*					1.25 [1.11-1.40]	1.63x10^{-4}	1.22 [1.09-1.38]	7.22x10^{-4}	1.21 [1.08-1.37]	1.26x10^{-3}	1.21 [1.08-1.36]	1.37x10^{-3}
	Alcohol drinker status	*Former*					1.14 [1.06-1.24]	1.10x10^{-3}	1.13 [1.04-1.23]	3.48x10^{-3}	1.11 [1.02-1.20]	1.38x10^{-2}	1.11 [1.02-1.20]	1.32x10^{-2}
	(ref. Current)	*Never*					1.10 [0.99-1.22]	7.29x10^{-2}	1.08 [0.98-1.20]	1.25x10^{-1}	1.07 [0.97-1.19]	1.69x10^{-1}	1.07 [0.97-1.19]	1.68x10^{-1}
	BMI (kg/m^2)	*[25.30[*					1.09 [0.93-1.29]	2.94x10^{-1}	1.05 [0.89-1.25]	5.60x10^{-1}	1.01 [0.85-1.20]	8.90x10^{-1}	1.01 [0.85-1.20]	8.99x10^{-1}
	(ref. <25)	*[30,40[*					1.24 [1.06-1.43]	5.29x10^{-3}	1.15 [0.98-1.35]	8.24x10^{-2}	1.04 [0.89-1.23]	6.06x10^{-1}	1.04 [0.89-1.23]	6.14x10^{-1}
		>=40					1.18 [1.08-1.29]	2.51x10^{-4}	1.13 [1.04-1.24]	6.82x10^{-3}	1.07 [0.97-1.17]	1.59x10^{-1}	1.07 [0.97-1.17]	1.61x10^{-1}
B	Cholesterol (mmol/L)								0.98 [0.84-1.14]	7.84x10^{-1}	1.11 [0.92-1.33]	2.84x10^{-1}	1.11 [0.92-1.33]	2.80x10^{-1}
	HDL cholesterol (mmol/L)								0.92 [0.76-1.11]	3.96x10^{-1}	0.91 [0.75-1.10]	3.19x10^{-1}	0.91 [0.75-1.10]	3.14x10^{-1}
	Triglycerides (mmol/L)								1.04 [0.90-1.20]	6.35x10^{-1}	0.99 [0.85-1.15]	8.74x10^{-1}	0.99 [0.85-1.15]	8.77x10^{-1}
	Vitamin D (nmol/L)								0.89 [0.77-1.03]	1.09x10^{-1}	0.91 [0.79-1.05]	1.91x10^{-1}	0.91 [0.79-1.05]	2.00x10^{-1}
	Cystatin C (mg/L)								1.10 [1.02-1.18]	8.32x10^{-3}	1.05 [0.97-1.14]	2.33x10^{-1}	1.05 [0.97-1.14]	2.39x10^{-1}
M	Cancer (ref. No)	*Yes*									1.01 [0.91-1.12]	8.69x10^{-1}	1.01 [0.91-1.13]	8.68x10^{-1}
	Cardiovascular (ref. No)	*Yes*									1.22 [1.10-1.36]	2.66x10^{-4}	1.22 [1.10-1.36]	2.63x10^{-4}
	Hypertension (ref. No)	*Yes*									1.22 [1.06-1.41]	7.17x10^{-3}	1.22 [1.05-1.41]	7.34x10^{-3}
	Diabetes (ref. No)	*Yes*									1.16 [1.06-1.26]	8.08x10^{-4}	1.16 [1.06-1.26]	8.44x10^{-4}
	Respiratory (ref. No)	*Yes*									1.09 [0.97-1.22]	1.34x10^{-1}	1.09 [0.97-1.22]	1.37x10^{-1}
	Autoimmune (ref. No)	*Yes*									1.14 [1.03-1.27]	1.50x10^{-2}	1.14 [1.03-1.27]	1.48x10^{-2}
	ACE inhibitors	*Yes*									0.99 [0.90-1.10]	9.23x10^{-1}	1.00 [0.90-1.11]	9.35x10^{-1}
	Angiotensin II RB	*Yes*									1.03 [0.94-1.12]	5.68x10^{-1}	1.03 [0.94-1.12]	5.62x10^{-1}
	Oral steroid	*Yes*									1.09 [1.03-1.16]	4.26x10^{-3}	1.09 [1.03-1.16]	4.22x10^{-3}
	Statin	*Yes*									0.93 [0.82-1.06]	2.89x10^{-1}	0.93 [0.82-1.06]	2.80x10^{-1}
E	NO_x (ug/m^3)												1.07 [0.86-1.33]	5.29x10^{-1}
	PM10 (ug/m^3)												1.08 [0.92-1.26]	3.33x10^{-1}
	PM2.5 (absorbance/m)												1.01 [0.85-1.20]	9.27x10^{-1}
	PM2.5 (ug/m^3)												0.94 [0.75-1.18]	5.90x10^{-1}

A

B

			Model 1		Model 2		Model 3		Model 4		Model 5		Model 6	
			OR	p-value	OR	p-value	OR	p-value	OR	p-value	OR	p-value	OR	p-value
D	Age (years)		2.62 [2.45-2.81]	1.99x10^{-163}	2.41 [2.20-2.64]	8.72x10^{-83}	2.46 [2.25-2.69]	3.71x10^{-85}	2.34 [2.14-2.56]	7.75x10^{-76}	2.10 [1.92-2.31]	3.97x10^{-56}	2.11 [1.92-2.31]	3.55x10^{-56}
	Sex (ref. Women)	*Men*	1.22 [1.16-1.28]	1.61x10^{-13}	1.25 [1.19-1.32]	1.19x10^{-16}	1.24 [1.17-1.31]	1.44x10^{-14}	1.19 [1.12-1.26]	2.46x10^{-8}	1.19 [1.12-1.27]	2.43x10^{-8}	1.19 [1.12-1.27]	2.21x10^{-8}
	Ethnicity (ref. White)	*Black*	0.99 [0.92-1.06]	6.80x10^{-1}	0.95 [0.88-1.02]	1.68x10^{-1}	0.95 [0.88-1.02]	1.67x10^{-1}	0.94 [0.88-1.01]	1.04x10^{-1}	0.94 [0.88-1.01]	1.16x10^{-1}	0.95 [0.88-1.02]	1.34x10^{-1}
		Other	0.91 [0.85-0.99]	2.51x10^{-2}	0.90 [0.83-0.97]	9.52x10^{-3}	0.89 [0.82-0.97]	4.56x10^{-3}	0.87 [0.80-0.94]	7.11x10^{-4}	0.87 [0.80-0.94]	7.46x10^{-4}	0.87 [0.81-0.95]	9.47x10^{-4}
S	Education	*Intermediate*			1.02 [0.95-1.08]	6.30x10^{-1}	0.99 [0.93-1.06]	8.73x10^{-1}	1.00 [0.94-1.07]	9.64x10^{-1}	1.00 [0.93-1.06]	9.38x10^{-1}	1.00 [0.93-1.06]	8.97x10^{-1}
	(ref. High)	*Low*			1.06 [1.00-1.11]	5.29x10^{-2}	1.03 [0.97-1.09]	3.35x10^{-1}	1.03 [0.97-1.09]	3.39x10^{-1}	1.02 [0.96-1.08]	5.08x10^{-1}	1.02 [0.96-1.08]	5.39x10^{-1}
	Accomm. (ref. House)	*Flat*			1.04 [0.99-1.09]	1.53x10^{-1}	1.03 [0.98-1.09]	2.31x10^{-1}	1.03 [0.98-1.08]	3.16x10^{-1}	1.03 [0.98-1.08]	2.69x10^{-1}	1.03 [0.98-1.09]	2.39x10^{-1}
	Own	*Own with a mortgage*			1.03 [0.96-1.11]	3.52x10^{-1}	1.01 [0.94-1.09]	7.40x10^{-1}	1.00 [0.94-1.08]	9.12x10^{-1}	0.99 [0.92-1.06]	7.85x10^{-1}	0.99 [0.92-1.06]	7.48x10^{-1}
	(ref. Own outright)	*Rent*			1.16 [1.10-1.22]	8.72x10^{-9}	1.11 [1.06-1.17]	5.56x10^{-5}	1.09 [1.04-1.15]	6.53x10^{-4}	1.07 [1.02-1.13]	9.26x10^{-3}	1.07 [1.02-1.13]	9.71x10^{-3}
	Number in household				0.97 [0.90-1.05]	4.38x10^{-1}	0.98 [0.91-1.05]	5.44x10^{-1}	0.98 [0.91-1.05]	5.17x10^{-1}	0.98 [0.91-1.05]	4.99x10^{-1}	0.98 [0.91-1.05]	5.04x10^{-1}
	Income (GBP)	*<18,000*			1.05 [0.99-1.11]	1.10x10^{-1}	1.03 [0.97-1.09]	3.03x10^{-1}	1.02 [0.96-1.07]	5.61x10^{-1}	1.01 [0.95-1.07]	7.82x10^{-1}	1.01 [0.95-1.06]	8.35x10^{-1}
	(ref. 18,000	*31,000-51,999*			0.90 [0.84-0.97]	4.42x10^{-3}	0.91 [0.85-0.98]	9.65x10^{-3}	0.92 [0.86-0.99]	1.73x10^{-2}	0.92 [0.86-0.99]	2.18x10^{-2}	0.92 [0.86-0.99]	2.52x10^{-2}
	to 30,999)	*>52,000*			0.93 [0.85-1.00]	5.96x10^{-2}	0.95 [0.87-1.02]	1.67x10^{-1}	0.95 [0.88-1.03]	2.59x10^{-1}	0.96 [0.89-1.04]	3.43x10^{-1}	0.97 [0.89-1.05]	4.14x10^{-1}
	Occup.	*Healthcare worker*			1.05 [0.98-1.13]	1.52x10^{-1}	1.05 [0.98-1.13]	1.82x10^{-1}	1.04 [0.97-1.12]	2.38x10^{-1}	1.04 [0.96-1.11]	3.28x10^{-1}	1.04 [0.96-1.11]	3.32x10^{-1}
	(ref. Employed	*Unemployed*			1.13 [1.06-1.19]	4.93x10^{-5}	1.11 [1.05-1.18]	2.83x10^{-4}	1.11 [1.04-1.17]	7.24x10^{-4}	1.08 [1.02-1.14]	1.05x10^{-2}	1.08 [1.02-1.14]	1.01x10^{-2}
	(Other))	*Retired*			1.07 [1.00-1.14]	5.02x10^{-2}	1.07 [1.00-1.15]	3.68x10^{-2}	1.07 [1.01-1.15]	3.33x10^{-2}	1.06 [0.99-1.13]	8.52x10^{-2}	1.06 [0.99-1.13]	8.31x10^{-2}
H R F	Smoking status	*Former*					1.09 [1.03-1.15]	2.63x10^{-3}	1.08 [1.03-1.15]	3.75x10^{-3}	1.06 [1.00-1.12]	4.37x10^{-2}	1.06 [1.00-1.12]	4.35x10^{-2}
	(ref. Never)	*Current*					1.25 [1.20-1.31]	2.10x10^{-21}	1.23 [1.17-1.29]	4.43x10^{-18}	1.21 [1.16-1.27]	7.91x10^{-16}	1.21 [1.16-1.27]	8.36x10^{-16}
	Alcohol drinker status	*Former*					1.04 [0.99-1.08]	9.46x10^{-2}	1.02 [0.98-1.07]	2.97x10^{-1}	1.01 [0.97-1.06]	5.38x10^{-1}	1.01 [0.97-1.06]	5.37x10^{-1}
	(ref. Current)	*Never*					1.09 [1.04-1.14]	1.07x10^{-4}	1.08 [1.03-1.12]	9.35x10^{-4}	1.07 [1.02-1.12]	2.37x10^{-3}	1.07 [1.02-1.12]	2.34x10^{-3}
	BMI (kg/m^2)	*[25.30[*					0.99 [0.93-1.05]	6.99x10^{-1}	0.98 [0.92-1.04]	4.80x10^{-1}	0.97 [0.91-1.03]	3.32x10^{-1}	0.97 [0.91-1.03]	3.24x10^{-1}
	(ref. <25)	*[30,40[*					1.10 [1.04-1.17]	1.05x10^{-3}	1.05 [0.99-1.12]	1.15x10^{-1}	1.01 [0.95-1.08]	7.93x10^{-1}	1.01 [0.94-1.08]	8.07x10^{-1}
		>=40					1.10 [1.06-1.15]	4.87x10^{-6}	1.06 [1.02-1.11]	6.79x10^{-3}	1.03 [0.98-1.07]	2.61x10^{-1}	1.02 [0.98-1.07]	2.74x10^{-1}
B	Cholesterol (mmol/L)								0.83 [0.78-0.89]	3.71x10^{-8}	0.89 [0.82-0.96]	2.73x10^{-3}	0.89 [0.82-0.96]	2.77x10^{-3}
	HDL cholesterol (mmol/L)								1.06 [0.99-1.14]	1.05x10^{-1}	1.06 [0.99-1.14]	1.13x10^{-1}	1.06 [0.99-1.15]	1.06x10^{-1}
	Triglycerides (mmol/L)								1.01 [0.94-1.07]	8.63x10^{-1}	0.99 [0.92-1.05]	6.66x10^{-1}	0.98 [0.92-1.05]	6.49x10^{-1}
	Vitamin D (nmol/L)								0.89 [0.84-0.94]	6.49x10^{-5}	0.90 [0.85-0.95]	2.88x10^{-4}	0.90 [0.85-0.95]	2.75x10^{-4}
	Cystatin C (mg/L)								1.15 [1.11-1.18]	3.29x10^{-22}	1.12 [1.08-1.15]	2.66x10^{-13}	1.12 [1.08-1.15]	2.42x10^{-13}
M	Cancer (ref. No)	*Yes*									1.23 [1.18-1.28]	2.29x10^{-23}	1.23 [1.18-1.28]	2.44x10^{-23}
	Cardiovascular (ref. No)	*Yes*									1.17 [1.12-1.23]	4.10x10^{-12}	1.17 [1.12-1.23]	4.47x10^{-12}
	Hypertension (ref. No)	*Yes*									1.01 [0.95-1.07]	7.59x10^{-1}	1.01 [0.95-1.07]	7.56x10^{-1}
	Diabetes (ref. No)	*Yes*									1.09 [1.05-1.14]	1.23x10^{-5}	1.09 [1.05-1.14]	1.21x10^{-5}
	Respiratory (ref. No)	*Yes*									1.15 [1.10-1.20]	1.80x10^{-9}	1.15 [1.10-1.20]	2.21x10^{-9}
	Autoimmune (ref. No)	*Yes*									1.06 [1.02-1.11]	7.71x10^{-3}	1.06 [1.02-1.11]	7.58x10^{-3}
	ACE inhibitors	*Yes*									1.07 [1.02-1.12]	2.85x10^{-3}	1.07 [1.02-1.12]	2.87x10^{-3}
	Angiotensin II RB	*Yes*									1.03 [0.99-1.07]	1.20x10^{-1}	1.03 [0.99-1.07]	1.18x10^{-1}
	Oral steroid	*Yes*									1.00 [0.97-1.04]	8.23x10^{-1}	1.00 [0.97-1.04]	8.24x10^{-1}
	Statin	*Yes*									0.93 [0.88-0.98]	1.11x10^{-2}	0.93 [0.88-0.98]	1.12x10^{-2}
E	NO$_x$ (ug/m^3)												1.05 [0.95-1.16]	3.66x10^{-1}
	PM10 (ug/m^3)												1.03 [0.96-1.10]	3.87x10^{-1}
	PM2.5 (absorbance/m)												0.93 [0.86-1.00]	5.74x10^{-2}
	PM2.5 (ug/m^3)												1.00 [0.91-1.11]	9.34x10^{-1}
